# Proceedings of the Indo-U.S. bilateral workshop on accelerating botanicals/biologics agent development research for cancer chemoprevention, treatment, and survival

**DOI:** 10.1002/cam4.42

**Published:** 2013-02-03

**Authors:** Nagi B. Kumar, Medha Dhurandhar, Bharat Aggarwal, Shrikant Anant, Kenyon Daniel, Gary Deng, Julie Djeu, Jinhui Dou, Ernest Hawk, B. Jayaram, Libin Jia, Rajendra Joshi, Madhuri Kararala, Devarajan Karunagaran, Omer Kucuk, Lalit Kumar, Mokenge Malafa, G. J. Samathanam, Fazlul Sarkar, Maqsood Siddiqi, Rana P. Singh, Anil Srivastava, Jeffrey D. White

**Affiliations:** 1 Moffitt Cancer Center, Tampa, Florida, 33612-9497; 2 Centre for Development of Advanced Computing, Pune UniversityPune, 411007, India; 3 The University of Texas, M.D. Anderson Cancer CenterHouston, Texas, 77054; 4 The University of Kansas Medical CenterKansas City, Kansas, 66160; 5 Memorial Sloan-Kettering Cancer CenterNew York, New York, 10021; 6 Food and Drug AdministrationSilver Springs, Maryland, 20993; 7 India Institute of Technology-DelhiNew Delhi, 110016, India; 8 National Cancer Institute, NIHBethesda, Maryland, 20892; 9 Bioinformatics Scientific and Engineering Computing, Pune UniversityPune, 411007, India; 10 University of MichiganAnn Arbor, Michigan, 48109-5930; 11 Department of Biotechnology, India Institute of Technology – MadrasChennai, 600036, India; 12 Emory Healthcare, The Emory Clinic Winship Cancer InstituteNE Atlanta, Georgia, 30322; 13 Institute Rotary Cancer Hospital (IRCH), All India Institute of Medical SciencesNew Delhi, 110029, India; 14 Department and Transfer DivisionDepartment of Science and Technology, Government of IndiaIndia; 15 Barbara Ann Karmanos Cancer InstituteDetroit, Michigan, 48201; 16 Cancer Foundation of IndiaTiljala, Kolkata, 700039, India; 17 School of Life Sciences, Central University of GujaratGujarat, 382030, India; 18 Open Health Systems Laboratory at Johns Hopkins Montgomery County CampusRockville, Maryland, 20850

**Keywords:** Biologics, botanicals, cancer, chemoprevention, drug development

## Abstract

With the evolving evidence of the promise of botanicals/biologics for cancer chemoprevention and treatment, an Indo-U.S. collaborative Workshop focusing on “Accelerating Botanicals Agent Development Research for Cancer Chemoprevention and Treatment” was conducted at the Moffitt Cancer Center, 29–31 May 2012. Funded by the Indo-U.S. Science and Technology Forum, a joint initiative of Governments of India and the United States of America and the Moffitt Cancer Center, the overall goals of this workshop were to enhance the knowledge (agents, molecular targets, biomarkers, approaches, target populations, regulatory standards, priorities, resources) of a multinational, multidisciplinary team of researcher's to systematically accelerate the design, to conduct a successful clinical trials to evaluate botanicals/biologics for cancer chemoprevention and treatment, and to achieve efficient translation of these discoveries into the standards for clinical practice that will ultimately impact cancer morbidity and mortality. Expert panelists were drawn from a diverse group of stakeholders, representing the leadership from the National Cancer Institute's Office of Cancer Complementary and Alternative Medicine (OCCAM), NCI Experimental Therapeutics (NExT), Food and Drug Administration, national scientific leadership from India, and a distinguished group of population, basic and clinical scientists from the two countries, including leaders in bioinformatics, social sciences, and biostatisticians. At the end of the workshop, we established four Indo-U.S. working research collaborative teams focused on identifying and prioritizing agents targeting four cancers that are of priority to both countries. Presented are some of the key proceedings and future goals discussed in the proceedings of this workshop.

## Introduction

Cancer is a leading cause of death worldwide [1] with deaths projected to continue to rise to over 13.1 million in 2030. Based on these projections, and in response to the call for action from the World Health Organization for a multistakeholder engagement [1], the ultimate goal of our group is to prioritize and continue to enhance international collaboration to promote and support the multidimensional and multisectoral research that is needed in order to generate or strengthen the evidence-based cancer prevention and control strategies [2,3].

Botanicals/biologics have been shown to influence multiple biochemical and molecular cascades that inhibit mutagenesis, proliferation, induce apoptosis, and suppress the formation and growth of human cancers, thus modulating several hallmarks of carcinogenesis. These agents appear promising in their potential to make a dramatic impact in cancer prevention and treatment, with a significantly superior safety profile than most agents evaluated to date [4–12]. However, it is clear that although several botanicals have been characterized and used for hundreds of years (Traditional Chinese Medicine, Ayurveda, Siddha, Unani) [3,13], there have been several challenges and limitations toward progress in this field. The slow pace of growth of several of these leads could be attributed to regulatory protection of classical formulation, lack of standardization, quality control, mechanism-based studies, population-based normal range of biomarkers, good laboratory practices, and translational scientists engaged in conducting well-designed trials. Similarly, in spite of the national commitment, there are only a few groups in the United States focused in systematic drug development using botanicals/biologics. There is, thus, an urgent need to pool resources and to bring together key stakeholders to find productive ways to systematically accelerate botanicals/biologics drug development for cancer chemoprevention and treatment. Groups working toward similar objectives could learn from one another's successes and failures, furthering progress toward a shared goal (Extending the Spectrum of Precompetitive Collaboration in Oncology Research – Workshop Summary Released: 22 July 2010, Institute of Medicine, USA) [2].

## Proceedings

The workshop opened with a keynote lecture focused on “Past Experiences and Lessons Learned from Definitive Chemoprevention Trials: What Went Wrong? Where Do We Go From Here? The identification of chemopreventive agents holds tremendous promise in reducing the burden of cancer. Past trials of preventive agents offer important lessons that can inform the design and conduct of future trials. Important lessons learned regarding agents come from ATBC [4] and CARET [5], which demonstrated the need for more preclinical and early-phase work before undertaking phase III trials; from BCPT [6] and STAR [7], which showed that safety can be improved in iterative generations of agents and trials; from the APC [8], FAP [9], and aspirin in adenoma prevention trials [10–12], which highlighted the benefit of preclinical and Phase II testing, as well as the imperative for broad, sensitive toxicological, and human safety assessments; and finally the DFMO [10] and Sulindac combination trial, which demonstrated that synergy between agents can lead to lower doses, improved efficacy, and fewer or less severe toxicities. Regarding cohorts, we have learned there are substantial benefits to employing germline, familial, or increased-risk cohorts, including, among others, more power over a shorter time frame. An assessment of endpoints in trials resulting in approval of a preventive agent reveals that nearly all have been approved on the basis of intraepithelial neoplasia, particularly in accessible organs. Lessons gleaned regarding the overall design of clinical trials underscore the importance of the randomized, placebo-controlled design and the need for long-term follow-up and monitoring to meet Food and Drug Administration requirements and promote acceptance in the marketplace. Applying these and other lessons to the design of future chemoprevention trials should facilitate the translation of novel preventive agents into the clinic (Table 1).

**Table 1:** Agenda of presentations and presenters at the workshop on botanical/biologics agent development

Presenter	Presentation
Bharat Aggarwal, Ph.D.	The Promiscuous Targeting of Multiple Signaling pathways by Botanical and Biologics – Vice or Virtue?
Shrikant Anant, Ph.D.	Approaches to development of Novel therapeutic agents based natural dietary compounds for Cancer Chemoprevention and Treatment
Dr. Kenyon Daniel, Ph.D.	Virtual Screening and Molecular Modeling for Drug Discovery
Dr. Gary Deng, MD, Ph.D.	Promises and Challenges of Establishing Collaborations with InternationalPartners for Cancer Drug Development: Our Experience
Dr. Medha Dhurandhar and Dr. Rajendra Joshi	Combinational analytical approaches for chemopreventive agent development deplying in silico technologies
Julie Y. Djeu, Ph.D.	Icarlin and its derivative, ICT, exert anti-inflammatory, antitumor effects, and modulate myeloid-derived suppressive cells (MDSCs) functions
Dr. Jinhui Dou, Ph.D.	FDA Policy on the US Clinical Trials for Botanical and Biologic Drugs – General versus Specific Guidelines
Shanker Gupta	Indo-U.S. Collaborative Research – Opportunities
Dr. Ernest Hawk, M.D., M.P.H.	Past Experiences – Lessons Learned (SELECT, CARET, Retinoids vs. Statins, COX-2 inhibitors): What Went Wrong? Where Do We Go From Here?
Dr. B. Jayaram	In Silico Databases as Sources for Chemical structures of Natural Products
Libin Jia, Ph.D.	National Commitment to Build Collaborations and Partnerships in Botanical and Biologics for Cancer
Professor Devarajan Karunagaran	Strategies to sensitize cancer cells to curcumin-induced apoptosis
Dr. Omer Kucuk, M.D.	Botanicals in Prostate cancer Prevention and Treatment
Lalit Kumar, Ph.D.	Cancer Chemoprevention and Transdisciplinary Research New Approaches in Cancer Drug Discovery
Dr. Nagi Kumar, Ph.D., R.D., F.A.D.A.	Clinical Trials in Prostate and Lung Cancer Chemoprevention
Dr. Mokenge Malafa, M.D.	Approach in Developing Botanicals for Pancreatic Cancer
Dr. Cathy Meade, Ph.D.	Other Critical Issues in Principles of Research
Jong Park, Ph.D.	Preclinical trials for evaluation of safety and effectiveness of botanicals for chemoprevention: The green tea experience
Sam Petroda	International Collaborations in cancer Research and Future Directions
Dr. G. J. Samathanam	Currently Funded Projects in the Indian Portfolio of International Collaborations in Cancer Research and Future Directions Indian Regulatory Policies that affect Botanical Drug Development
Dr. Fazlul Sarkar, Ph.D.	Nutraceutical Research: Bench to Clinic
Dr. Michael Schell, Ph.D.	Improved Design of Clinical Trials with Botanicals and Biologics: Statistical Considerations
Professor Maqsood Siddiqi	Botanicals for Cancer Treatment and Prevention
Professor Rana P. Singh	Multiple Targets of Phytochemicals in Cancer Chemoprevention - Cell Cycle, Apoptosis and Angiogenesis
Anil Srivatsava	Model for the future
Dr. Jeffrey D. White, M.D.	Currently Funded RO1s, PO1s, R21s, and R03s in Our Portfolio Communication Models-Problems and Solutions Resources in the United States (Laboratory/Clinical)

This session was followed by the current approaches for screening agents for drug development. Adoption of computational methods to discover new drugs has recently experienced a true renaissance with several new and exciting techniques being developed and currently employed to design new drug candidates and to rapidly bring these agents to the clinic at relatively lower costs. Traditional approaches to drug discovery have involved target identification and validation and lead identification and optimization. However, in the past decade, several contemporary approaches and enabling technologies like High-Performance Computing, Grid Computing, and Cloud Computing have evolved. Virtual screening and molecular modeling for drug discovery including grid-based docking, quantum mechanics, molecular mechanics, molecular dynamics, normal-mode vibration, and mutational analysis were discussed. For complex diseases like cancer, traditional methods of targeting a single protein is found profoundly insufficient laying the foundation for polypharmacological and combinatorial analysis harnessing in silico techniques viz. Molecular Topology, Network Pharmacology, and Combinatorial Chemistry. A network perspective of complex cancers has direct implications in the drug discovery process as it changes the target entity from a single protein to entire molecular pathways and/or cellular networks. Examples of success included the work of the Bioinformatics Group at the Centre for Development of Advanced Computing (India) in collaboration with cancer Biomedical Informatics Grid (caBIG^®^; National Cancer Institute, National Institute of Health, Rockville, Bethesda, Maryland, USA), which has developed a grid-enabled web-based automated pipeline, as well as homology-based prediction of protein structures, with an emphasis on cancer-related proteins. Current drug design software also falls short of expectations even if the structures of drug targets are known [4–6,13]. Addressing these issues from a physico-chemical perspective, an approach whereby the development of all atom energy-based methodologies for whole-genome analysis (ChemGenome, National Human Genome Research Institute, National Institutes of Health, Bethesda, Maryland, http://www.genome.gov), tertiary structure prediction of proteins (Bhageerath and Bhageerath-H), and protein/DNA-targeted lead molecule design (Sanjeevini) was discussed. These methods can be configured into an assembly line to deliver hit molecules from genomic information. The software and a host of other utilities are freely accessible to the global user community (http://www.scfbio-iitd.res.in) [14–18]. It was evident that the availability of contemporary software applications and infrastructure for collaborative research in both India and the United States was outstanding and ready for application.

This session was followed by presentations of a systematic approach using the traditional scientific paradigm to accelerate agent development using botanicals/biologics for cancer prevention and treatment in both countries. As cancers are caused by perturbations of multiple signaling pathways, the value of promiscuous targeting of botanicals/biologics was presented compared with mono-targeted “smart drugs,” using curcumin as an example of an agent that targets multiple signaling pathways [19–21]. Additional studies demonstrating a protective role of curcumin in arsenic-induced lymphocyte DNA damage with implications as an effective approach to overcome arsenic toxicity and its consequential adverse health effects in arsenic-exposed human populations were presented [22]. Early work on several multitargeting, novel botanicals such as limonoids from the neem tree (*Azadirachta indica*) was reported with research demonstrating that both azadirachtin and nimbolide significantly suppressed the viability of HeLa cells in a dose-dependent manner by inducing cell cycle arrest at G0/G1 phase accompanied by p53-dependent p21 accumulation and downregulation of the cell cycle regulatory proteins cyclin B, cyclin D1, and PCNA [23]. Similarly, nimbolide, a neem-derived tetranortriterpenoid was shown to concurrently abrogate canonical NF-κB and Wnt signaling and induce intrinsic apoptosis in HepG2 cells [24]. Quercetin exerts opposing effects on different signaling networks to inhibit cancer progression, and thus, it is a classic candidate for anticancer drug design [25,26]. Other examples included 3, 5, 7-trihydroxy-4′-methoxy-8-(3-hydroxy-3-methylbutyl)-flavone (ICT), a novel derivative of Icariin (ICA), the major active ingredient of Herba Epimedii. ICA and, more robustly, ICT directly modulate MDSC signaling, and therefore altered the phenotype and function of these cells, both in vitro and in vivo. Icariin mediated the anti-inflammatory functions including downregulation of TNF-α, PGE_2_, and nitric oxide and inhibition of NF-κB p65 activation. Decreased expression of S100A8/9 observed and inhibition of activation of STAT3 and AKT may in part be responsible for the observed results [27].

Presentations that followed focused on elucidating the molecular mechanism of action of several botanicals/biologics in preclinical studies and provided examples of subsequent clinical trial results on human breast, prostate, colon, and pancreatic cancer patients. The potential mechanisms of action of lycopene include its antioxidant and anti-inflammatory, antiproliferative effects as well as modulation of gene expression through epigenetic effects [28,29]. Isoflavones, a potent proteasome inhibitor [30], produced moderate modulation of steroid hormones and stabilization or reduction of serum prostate-specific antigen and reduced percentage of cells expressing Ki-67 posttreatment [31,32] with no toxicity [33]. It was also observed that genistein downregulates androgen receptor (AR) expression and produces an increase in FOX01 activity, a pathway that may be more relevant in African American (AA) men [34]. Significant increase in NF-κB activity was reported in prostate cancer cells exposed to chemotherapy and radiation. However, pretreatment with genistein completely abrogated the chemotherapy and radiation induced increases in NF-κB. Prostate cancer patients who received external beam radiation therapy who received isoflavones demonstrated significant reductions in radiation-induced toxicity to normal tissue structure, improvement in the erectile dysfunction, and urinary continent function [35]. Novel preclinical data on the combination of isoflavone with conventional therapeutics in pancreatic cancer demonstrating safety were presented [36].

Decursin, a novel coumarin compound, strongly inhibits growth and induces death in human prostate carcinoma DU145, PC-3, and LNCaP cells [37,38]. Report of findings revealed that the novel anticancer effects of decursin were mediated via induction of antiangiogenesis, and cell cycle arrest and apoptosis selectively in human prostate cancer cells. Preclinical and clinical trial results on 3, 3′-diindolylmethane (DIM) demonstrated that miR-34a is typically silenced through methylation in prostate cancer; however, BR-DIM intervention resulted in the demethylation of miR-34a promoter, resulting in its re-expression, which led to the downregulation of AR expression, one of the target genes of miR-34a [39]. Green tea polyphenols was shown to selectively inhibit the proteasome activity in intact human prostate cancer cells and consequently accumulates IkB-α and p27 proteins, leading to growth arrest [40], providing the rationale for a phase II clinical trial for prostate cancer prevention [41]. Tocotrienols inhibited NF-κB activity and the survival of human pancreatic cancer cells in vitro and in vivo, including observations that the bioactivity of the four natural tocotrienol compounds (α-, β-, δ-, and γ-tocotrienol) was directly related to their ability to suppress NF-κB activity in vitro and in vivo. The most bioactive tocotrienol for pancreatic cancer, δ-tocotrienol, significantly enhanced the efficacy of gemcitabine to inhibit pancreatic cancer growth and survival in vitro and in vivo and associated with significant suppression of NF-κB activity and the expression of NF-κB transcriptional targets [42].

Attempts to understand the cellular origin of cancer have advanced the theory of cancer stem cells (CSCs). These rare CSCs have indefinite proliferative potential and are believed to be responsible for tumor invasiveness and heterogeneity. Research approaches and rationale focused on identifying botanicals/biologics for prevention and therapy that target the stem cells were presented [43]. The CSC hypothesis asserts that malignancies arise in tissue stem and/or progenitor cells through the dysregulation or acquisition of self-renewal [44]. In a study to determine whether the dietary polyphenols, curcumin, and piperine are able to modulate the self-renewal of normal and malignant breast stem cells, the effects of these compounds on mammosphere formation, expression of the breast stem cell marker aldehyde dehydrogenase (ALDH), and Wnt signaling were examined. Results demonstrated that curcumin and piperine separately, and in combination, inhibit breast stem cell self-renewal but do not cause toxicity to differentiated cells [45]. Stem cell signaling pathways, self-renewal, epigenetics of stem cell regulatory elements could be used as efficacy surrogate biomarkers in clinical trials of both cancer preventive and treatment compounds [46]. It was evident from these presentations and discussions that the current and ongoing research in this field was substantial, with a promise of several novel agents in the pipeline, poised to be evaluated in phase I–III clinical trials to ultimately reach the patient's bedside.

Representatives from the regulatory bodies from India and United States discussed potential challenges and successes for international collaborative research. FDA, USA, published a draft Guidance for Industry-Botanical Drug Products (Guidance) in 2000 and finalized the Guidance in 2004, to illustrate the current thinking on the development of botanical drugs. The FDA Policy on the U.S. Clinical Trials for Botanical and Biologic Drugs and the Investigational New Drug Approval process was presented. Representatives from the Science and Technology Forum (India) described that the efforts are ongoing to uplift the infrastructure development and international harmonization of laboratories R&D through GLP, National Accreditation Board of Testing and Calibration of Laboratory, Metabolic Wards for GCP, Revision of Ethical Guidelines for Biomedical Research on Human and Animal research and Clinical Trial Registry (CTRI), and it is mandatory according to Drugs Controller General India that all the clinical trials undertaken in India need to be registered with CTRI. Examples of experience working with regulatory agencies and international pharmaceutical companies in early phase I–II clinical trials using agents' ranges from highly defined extract from Maitake mushroom, crude extract from a single herb *Coptis sinensis*, and two complex herbal formulations requiring investigator-initiated IND approvals from FDA were presented [47,48]. Building a team that combines complementary expertise, communicates well, and ensures development of personal relationships was critical for success with international collaborations. Other critical issues discussed included statistical considerations, cultural, literacy, economic, and social considerations when designing clinical trials.

It was reported that approximately $114 million of an NCI budget of $5 billion directly supports complementary and alternative medicine (CAM) research, including research of botanicals and botanical-related products. In fiscal year (FY) 2010, over 340 of grants funded by NCI supported CAM research, and about 15% of these supported botanical or botanical-related research [49]. The Office of Cancer Complementary and Alternative Medicine (OCCAM) of the NCI is working to build a research portfolio with areas of special interest that includes identifying Novel Therapeutics from the Pharmacopeia of Traditional Medical Systems and provided examples of a funded trial of Chinese Herbal Medicine PHY906 [50,51] as a Novel Paradigm for Cancer Chemotherapy. In India, the primary funding groups include the Indo-U.S. Science and Technology Forum with support from Central, State government agencies, and private foundations.

## Future Directions

On the final day of the workshop, four collaborative working groups focused on initiating collaborative research projects using the traditional and novel funding mechanisms in both countries were established. We plan to establish a joint repository of botanicals/biologics ready for prioritization for preclinical and clinical trials, targeting major cancers in both countries. Guided by an independent advisory board of stakeholders from India and the United States, the group continues to communicate regularly with future meetings scheduled during national and international scientific conferences. It is clear that both countries have their strengths and resources, which when combined can actively facilitate the building of an interdisciplinary community harnessing system of Information and Communications Technology toward accelerating botanicals and biologic drug development on an international scale for cancer prevention and treatment. Of significant importance is also the impact this approach is likely to have on the economics of drug discovery. We predict that this collaborative effort can result in research breakthroughs, which will not only bring new hope but also create a new class of anticancer drugs that will help millions of cancer patients and those at high risk for this disease in both our countries and will benefit the world population at large.
